# Facile Synthesis of Molecularly Imprinted Ratiometric Fluorescence Sensor for Ciguatoxin P-CTX-3C Detection in Fish

**DOI:** 10.3390/foods11203239

**Published:** 2022-10-17

**Authors:** Zhenke Qi, Cheng Xiang, Xingguo Tian, Xiaoyan Xu

**Affiliations:** Guangdong Provincial Key Laboratory of Food Quality and Safety, College of Food Science, South China Agricultural University, Guangzhou 510642, China

**Keywords:** ratiometric fluorescence sensor, molecular imprinting, fragmentary dummy template, carbon dots, P-CTX-3C

## Abstract

Ciguatoxin (CTX) detection methods are essential due to the serious hazard that bioaccumulation in fish and transmission along the food chain poses to human health. We report the rapid and simple development of a dual-emitting, molecularly imprinted, ratiometric fluorescence sensor (MIPs@BCDs/RCDs@SiO_2_) to detect ciguatoxin P-CTX-3C with high sensitivity and selectivity. The sensor was fabricated via sol–gel polymerization using monensin as the fragmentary dummy template molecule, blue carbon dots (BCDs) as the response signal, and red carbon dots (RCDs) as the reference signal. The fluorescence emission of BCDs was selectively quenched in the presence of P-CTX-3C, leading to a favorable linear correlation between the fluorescence intensity ratio (I_440_/I_675_) and the P-CTX-3C concentration in the range of 0.001–1 ng/mL with a lower detection limit of 3.3 × 10^−4^ ng/mL. According to LC-MS measurement results, the proposed sensor can rapidly detect ciguatoxin P-CTX-3C in coral reef fish samples with satisfactory recoveries and standard deviations. This study provides a promising strategy for rapid trace analysis of marine toxins and other macromolecular pollutants in complex matrices.

## 1. Introduction

Ciguatoxins (CTXs) are a class of polyether marine algal toxins produced by microalgae of the genera *Gambierdiscus* and *Fukuyoa*, which can accumulate in coral reef fish such as grouper, perch, and eel [1]. CTXs, particularly P-CTX-3C, can competitively bind to voltage-gated sodium and potassium channels. This interference triggers ciguatera poisoning (CP), causing gastric, neurological, and cardiovascular diseases in humans when seafood containing CTXs is ingested [2]. At least 50,000 to 100,000 people suffer from CP every year [3]. The U.S. Food and Drug Administration recommends that CTXs in fish should be limited to 0.01 μg/kg [4]. The methods commonly adopted for CTX detection are cytotoxicity tests [5], receptor binding tests [6], and liquid chromatography–mass spectroscopy (LC-MS) [7]. The sensitivity and accuracy of the first method are excellent, but it is not able to discriminate between different CTX analogues. LC-MS has the advantages of specificity and sensitivity, but the method is expensive and requires trained personnel. The challenges in detecting CTXs are represented by their low concentration in natural samples, the matrix in which they can be found, and the presence of unknown/not yet described analogues. Therefore, it is of utmost importance to establish a simple, rapid, selective analytical method to detect CTXs in complex natural matrices.

Over the past decade, fluorescence sensors have been considered for the analysis of trace substances worldwide since they are highly sensitive, easy to use, and require short incubation time [8]. However, single-wavelength-based fluorescent sensors are susceptible to minute variations in instrument efficiency, probe concentration, environmental conditions, and excitation intensity. These vacillations reduce the accuracy and reliability of the detection [9,10]. In contrast, ratiometric fluorescence sensors, which use ratios between fluorescence intensities at two or more well-resolved wavelengths as the response signal, are inherently self-correcting. Thus, they effectively resolve the fluorescence vacillation problem [11,12]. Currently, most reported ratiometric fluorescence sensors are constructed using quantum dots, metal nanoclusters, organic dyes, or carbon nanomaterials as fluorophores [13,14,15,16]. Among these fluorescent materials, carbon dots (CDs) may be the most promising because of their considerable advantages: negligible toxicity, excellent biocompatibility, high photo and chemical stability, cost-effectiveness, and easy synthesis [17,18,19]. CDs are a class of multicolor photoluminescent (PL) materials that can be excited by a single wavelength [20]. Benefiting from such unique and outstanding features, dual-emitting ratiometric fluorescent sensors based on CDs could be developed for biological and chemical sensing.

A ratiometric fluorescent sensor’s specific recognition elements must detect the target analyte with high selectivity and without cross-reactivity from other compounds that can be present in natural samples [21,22]. The features of molecularly imprinted polymers (MIPs) make them suitable candidates for the development of chemical sensors. Additionally, they are customizable, stable, economic, and easy to prepare [23,24,25].

At present, several different methods are available to synthesize MIPs, including bulk polymerization, in situ polymerization, precipitation polymerization, suspension polymerization, swelling polymerization, and sol–gel polymerization [26,27]. However, some molecules are not suitable as templates for direct molecular imprinting due to their large molecular structures, strong toxicities, and/or high prices. These constraints have compelled researchers to break from convention and introduce a novel concept: a dummy template method based on fragmentary molecules [28,29]. For instance, Matsui et al. reported the construction of synthetic polymer receptors selective for atrazine via molecular imprinting. They used nonagrochemical trialkylmelamines as template molecules in place of atrazine, thus avoiding the troubles caused by analyte contaminants [30]. The authors proposed a fragment template docking molecular imprinting strategy for Microcystin-LR detection using L-leucine as the dummy template molecule [31].

Inspired by the studies mentioned above, we undertook the quick and simple construction of a novel molecularly imprinted ratiometric fluorescence sensor with P-CTX-3C adsorption specificity and fluorescent sensing capability via sol–gel polymerization. P-CTX-3C is not only an expensive analyte but also an extremely toxic one with a high risk of leakage, making it unfit as a template in MIP preparation. The sensor was therefore designed using monensin as the fragmentary dummy template, 3-aminopropyltriethoxysilane (APTES) as the functional monomer, tetraethylorthosilicate (TEOS) as the crosslinker, and two different colors of CDs, namely red carbon dots (RCDs) and blue carbon dots (BCDs), as the reference signal and the response signal, respectively. The obtained fluorescence sensor was well characterized, and the recognition and quenching properties were tested. The proposed molecular imprinting ratiometric fluorimetry method was successfully developed and tested for the detection of P-CTX3C traces in fish samples.

## 2. Materials and Methods

### 2.1. Reagents and Materials

3-(2-aminoethylamino)propyldimethoxymethylsilane (AEAPMS), TEOS, APTES, and monensin were purchased from Shanghai Macklin Biochemical Co., Ltd. (Shanghai, China). The standard analyte P-CTX-3C was purchased from Wako Pure Chemical Industries (Osaka, Japan).

### 2.2. Apparatus

High-resolution transmission electron microscope (HRTEM) images were obtained using a Talos F200S microscope with an acceleration voltage of 200 kV (Thermo Fisher Scientific, Waltham, MA, USA). TEM images were obtained using an FEI/Talos L120C microscope with an acceleration voltage of 200 kV (Thermo Fisher Scientific). X-ray photoelectron spectra were obtained using a 250Xi X-ray photoelectron spectroscopy (XPS) device with a testing temperature of −400 °C (Thermo Fisher Scientific). Fourier transform infrared (FT-IR) spectra ranging from 450 to 4000 cm^−1^ in KBr were measured with an FT-IR spectrometer (Bruker, Ettlingen, Germany). Ultraviolet–visible (UV–Vis) absorption spectra were acquired using a UV-3600 spectrometer (Shimadzu, Kyoto, Japan), and the fluorescence spectra were measured using a 970CRT spectrophotometer (INESA, Shanghai, China). The quantum yield of RCDs was obtained using an FLS1000/FS5 steady-state and transient-state fluorescence spectrometer (Edinburgh Instruments, Edinburgh, U.K.). The MIPs@BCDs/RCDs@SiO_2_ was purified using the KQ-100DE Ultrasonic Cleaner (Supmile, Jiangsu, China) with ultrasonic frequency of 40 KHz and the TG16WS High-Speed Centrifuge (XIANGZHI CENTRIFUGE, Hunan, China) with maximum rpm of 16,000 r/min. The obtained MIPs@BCDs/RCDs@SiO_2_ was dried using a (YIHENG Instruments, Shanghai, China) DZF-6050 vacuum oven. LC-MS analyses were caried out using a 1290-6470 ultra-high-performance liquid chromatograph/triple quadrupole tandem mass spectrometer (Agilent, Santa Clara, CA, USA).

### 2.3. Synthesis of Blue Carbon Dots

Under high-temperature heating, anhydrous citric acid was pyrolyzed and carbonized to form a nucleus; AEAPMS passivated and modified the nucleus’s surface to produce BCDs. The method used in this experiment improved upon the previously reported one-step hydrothermal method [32]. First, 10 mL of AEAPMS was added to a 150 mL three-necked flask, and the reaction system was degassed with nitrogen for 10 min and heated to 200 °C. Next, 0.5 g of anhydrous citric acid was fully dissolved in 3 mL of anhydrous ethanol under sonication. The mixed solution was quickly injected into the reaction system at a temperature of 200 °C under strong stirring for 1 min. The resulting product was centrifuged at 12,000 rpm for 30 min, and the supernatant was passed through a 0.22 μm organic membrane several times to remove unreacted chemicals. The product was then washed with petroleum ether three times to remove chemical substances of low polarity. Then, 1 mL of the purified product was dispersed in 9 mL of an absolute ethanol solution and stored at 4 °C in darkness.

### 2.4. Synthesis of Red Carbon Dots

RCDs were synthesized by extracting from Osmanthus fragrans according to reported methods [33]. Only fine leaves were selected. The petiole was cut off, and the leaves were washed repeatedly with deionized water to remove residual soluble impurities. Deionized water on the leaves evaporated to dryness at room temperature. The dried leaves were ground to powder for 30 min drastically and stored in dry conditions before use. Then, 1.0 g of leaf powder was added to 60 mL of an anhydrous ethanol solution and stirred vigorously at room temperature under dark conditions for 48 h to extract the crude product. After extraction, the crude product was centrifuged at 5000 rpm for 10 min and filtered through a 0.22 μm organic membrane to remove unreacted chemicals. The obtained nitrogen-doped RCDs appeared light green under visible light. They were transferred to a glass culture dish and dried in a vacuum drying oven at 40 °C for 8 h to obtain green powder samples, which were stored in dark conditions at 4 °C for later use.

### 2.5. Fabrication of Molecularly Imprinted Ratiometric Fluorescence Sensor

Silicon-coated RCDs (RCDs@SiO_2_) were prepared by the improved Stöber method [34]. First, 20 mg of RCDs powder was homogeneously dispersed in 10 mL of anhydrous ethanol by ultrasonic mixing. Subsequently, under stirring, 1 mL of TEOS was added to the mixed solution drop by drop at room temperature. After 30 min of mixing, 200 μL of 25% ammonia solution was added, followed by stirring at room temperature for 24 h. The obtained product was centrifuged and then washed sequentially with ethanol and deionized water to remove unreacted substances prior to drying in an oven at 40 °C for 48 h for later use.

The molecularly imprinted ratiometric fluorescence sensor (MIPs@BCDs/RCDs@SiO_2_) was fabricated via the improved sol–gel method [35]. First, 20 mg of RCDs@SiO_2_ and 0.5 mL of BCDs were uniformly dispersed in a 50 mL beaker containing 20 mL of anhydrous ethanol. Then, 20 mg of monensin and 140 μL of APTES were added to the mixed solution, and the reaction system was stirred vigorously for 1 h at room temperature to pre-polymerize. Subsequently, 260 μL of TEOS and 150 μL of 25% ammonia were added as a continuous stream of droplets under strong stirring. Afterward, the beaker was sealed with a molecular membrane and then stirred in dark conditions at room temperature for 24 h. Finally, the crude products were repeatedly ultrasonic centrifuged at 40 KHz and 10,000 rpm and washed with ethanol. The obtained products were dried overnight in a vacuum drying oven at 60 °C and then stored in dark conditions at 4 °C for later use. A non-imprinted ratiometric fluorescence sensor (NIPs@BCDs/RCDs@SiO_2_) was obtained via the same method with no monensin.

### 2.6. Fluorescence Measurements

All fluorescence intensity tests were carried out by using a fluorescence spectrophotometer under constant conditions. The light source was a 150 W xenon lamp, and the scanning sensitivity was set at level 3 in the range of 1–8. (The scanning sensitivity range of 1–8 means that the scanning intensity of the sample is gradually enhanced by the fluorophotometer, and the range of the fluorescence intensity of the fluorescence spectrum is 0–1000. Basically, the larger the peak value of the scanned fluorescence intensity spectrum within 1000, the better the peak shape. Thus, the setting of scanning sensitivity at level 3 was the optimum through a repeated testing.) The scanning slits for excitation and emission were 10 nm. First, to explore the effect of MIPs@BCDs/RCDs@SiO_2_ on different concentrations of P-CTX-3C, 100 μL of different concentrations of P-CTX-3C were added to 300 μL of 0.1 mg/mL MIPs@BCDs/RCDs@SiO_2_, obtaining final concentrations ranging from 0 ng/mL to 1.5 ng/mL. Afterwards, emission intensities at wavelengths ranging from 400 to 700 nm were measured at the excitation wavelength of 360 nm. Finally, fluorescence emission spectra were obtained, and the adsorption and sensing properties of the P-CTX-3C were analyzed. The fluorescence intensities of NIPs@BCDs/RCDs@SiO_2_ were tested following the same procedure.

### 2.7. Analysis of Reproducibility

Firstly, to conduct the adsorption effect experiment, 1 mL of 0.1 ng/mL P-CTX-3C was obtained and added to 3 mL of 0.1 mg/mL MIPs@BCDs/RCDs@SiO_2_ in a 10 mL centrifuge tube, which was fixed and shaken by the thermostatic oscillator with the oscillating time of 10 min, speed of 200 rpm, and temperature of 25 °C. The adsorption effect was evaluated by measuring the fluorescence quenching efficiency using a fluorescence spectrophotometer. Afterwards, the absorbed MIPs@BCDs/RCDs@SiO_2_ was eluted by absolute ethanol and reprepared into 3 mL of 0.1 mg/mL MIPs@BCDs/RCDs@SiO_2_, following the steps in the first procedure. Finally, the fluorescence quenching efficiency was obtained to evaluate the reproducibility of MIPs@BCDs/RCDs@SiO_2_ through four cycles of absorption and desorption of P-CTX-3C. Each cycle was repeated three times.

### 2.8. Analysis of Selectivity and Anti-Interference Ability

The analysis of selectivity was as follows. Firstly, 100 μL of 0.1 ng/mL of six structural analogs, namely monensin, P-CTX-3C, nigeriamycin, rotenone, ionomycin A, and lysozyme, were added to 300 μL of 0.1 mg/mL MIPs@BCDs/RCDs@SiO_2_ solution. Subsequently, the mixture was fixed in the thermostatic oscillator to shake for 10 min with the oscillating speed of 200 rpm and the temperature of 25 °C. The fluorescence intensities of each mixture were measured and recorded three times. To explore the differences in adsorption properties of these molecules to MIPs and NIPs, the same adsorption experiment was conducted for NIPs@BCDs/RCDs@SiO_2_. 

The anti-interference effect experiment was conducted with the following amino acids and ions. Firstly, 100 μL of 0.1 ng/mL P-CTX-3C and 100 μL of 0.2 μmol/L glutamic acid (Glu), asparagine (Asp), leucine (Leu), lysine (Lys), arginine (Arg), K^+^, Na^+^, Ca^2+^, Mg^2+^, Al^3+^, Hg^2+^, Cu^2+^, Fe^3+^, and Ag^+^ were added to 300 μL of 0.1 mg/mL MIPs@BCDs/RCDs@SiO_2_. Subsequently, the mixture was fixed in the thermostatic oscillator to shake for 10 min with the oscillating speed of 200 rpm and the temperature of 25 °C. The fluorescence intensities of each mixture were measured and recorded three times. To explore the difference in the anti-interference effect without P-CTX-3C, the same experiment was conducted adding 100 μL of anhydrous methanol instead of P-CTX-3C.

### 2.9. Analysis of Original and Spiked Samples

The fish samples—eel, bass, and grouper—were purchased from a market in Guangzhou, China. All the samples originated from Zhuhai, Guangdong. The average length and weight of eels, bass, and groupers were 82 cm, 3.3 kg; 35 cm, 1.8 kg; and 38 cm, 2.9 kg, respectively. We weighed 5 g of each sample and further crushed it into a 50 mL polypropylene centrifuge tube. Then, 15 mL of 99.9% anhydrous methanol was added to each sample and gradually mixed in over the course of 1 min. Each sample was extracted in an oscillator at 210 rpm for 15 min, followed by a centrifuge step at 14,000 rpm at 4 °C for 5 min. The residues were further extracted with the supernatant removed. The residues were evaporated to dryness at 40 °C in vacuum conditions. The obtained residues were dissolved in 5.0 mL of 99.9% anhydrous methanol and 4.5 mL of deionized water and added to a C18 column activated with 15 mL of 99.9% acetonitrile, 15 mL of 99.9% anhydrous methanol, and 10 mL of deionized water. They were then washed with 6.5 mL 65% methanol and drained. Afterward, they were eluted with 15 mL 99.9% acetonitrile, followed by drying completely at 40 °C in vacuum conditions. The dried products were then dispersed in 1 mL of a 99.9% methanol solution to obtain 5 g equivalents of fish flesh per mL. The obtained mixture was filtered with a millipore organic membrane of a needle filter (0.22 μm pore size, 33 mm diameter) and the filtrate was collected for analysis.

Firstly, 100 μL of the filtrate of three samples was added to 300 μL MIPs@BCDs/RCDs@SiO_2_ and absorbed by constant oscillation for 10 min, and the fluorescence intensities of the mixture were measured and recorded. Subsequently, the spiked and recovery experiments were conducted by adding 100 μL of 0.025 ng/mL, 0.25 ng/mL, and 0.75 ng/mL of P-CTX-3C standard solution to filtrate of three samples, and each sample was analyzed three times. Furthermore, the P-CTX-3C contents in the above sample solutions were also analyzed by LC-MS.

LC-MS analyses were caried out as described previously with modifications [36]. For the high-performance liquid chromatography, 20 μL of the filtrate was injected into a C18 column (4.6 × 250 mm id, 5 μm) at 30 °C with the velocity of flow of 0.25 mL/min. The eluate solution A was methanol containing 10 mM ammonium acetate and 0.1% formic acid, and the eluate solution B was 10 mM ammonium acetate and 0.1% formic acid. The initial solvent composition was 10% B with a linear gradient to 40% B at 3.0 min, ramped up to 50% B at 3.5 min, and a linear gradient to 80% B at 4.5 min, ramped up to 95% B at 7.0 min and maintained at 95% B between 7.0 and 8.0 min. For the mass spectrometer, the target toxins were ionized with electron spray ionization (ESI) equipped with positive ions and monitored with a multiple reaction monitoring (MRM) mode. Other settings were capillary voltage of 2.8 kV, cone voltage of 28 V, source temperature of 120 °C, nitrogen gas desolvation flowrate of 450 L/h at 350 °C, and cone gas of 50 L h^−1^, and when the qualitative ion pair was set to 1042.0, 1060.0, and 1078.0, the collision voltage was 18 V, 15 V, and 13 V, respectively.

## 3. Results and Discussion

### 3.1. Fabrication and Characterization of the Ratiometric Fluorescence Sensor

**Scheme 1:**
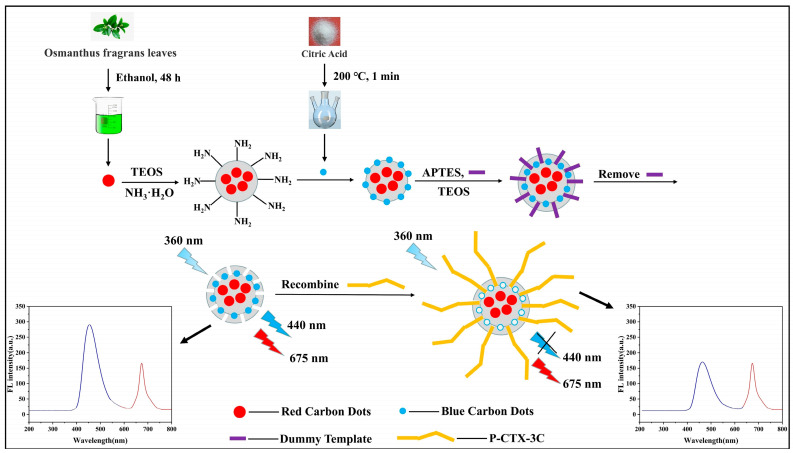
Schematic illustration of preparation procedure for MIPs@BCDs/RCDs@SiO_2_ sensor and recognition for P-CTX-3C.

Scheme 1 illustrates the preparation procedure of the ratiometric fluorescence sensor. As shown, BCDs and RCDs were synthesized from different carbon sources. RCDs@SiO_2_ was then prepared via the classic Stöber method. TEOS hydrolysis and ammonia catalysis caused RCDs to be wrapped with a silica shell. This shell prevented direct contact between RCDs and external P-CTX-3C, thus offering a constant reference signal for ratiometric detection. Subsequently, the improved sol–gel approach was undertaken to fabricate the molecularly imprinted ratiometric fluorescence sensor (MIPs@BCDs/RCDs@SiO_2_). Specifically, MIPs were prepared using a dummy monensin template, a fragment of the P-CTX-3C molecule. Afterward, the Si-O-Si chain between RCDs@SiO_2_ and MIPs was cross-linked and polymerized. Because the BCDs with surface passivation contained silicon groups, the Si-O-C bond and Si-O-R bond in each BCDs could cross-link with the Si-O-Si chain of the MIPs and further integrate into the MIPs structure. In the absence of monensin, the resultant ratiometric sensor MIPs@BCDs/RCDs@SiO_2_ exhibited two well-resolved emission peaks at 440 nm and 675 nm when excited by a single 360 nm excitation.

**Figure 1:**
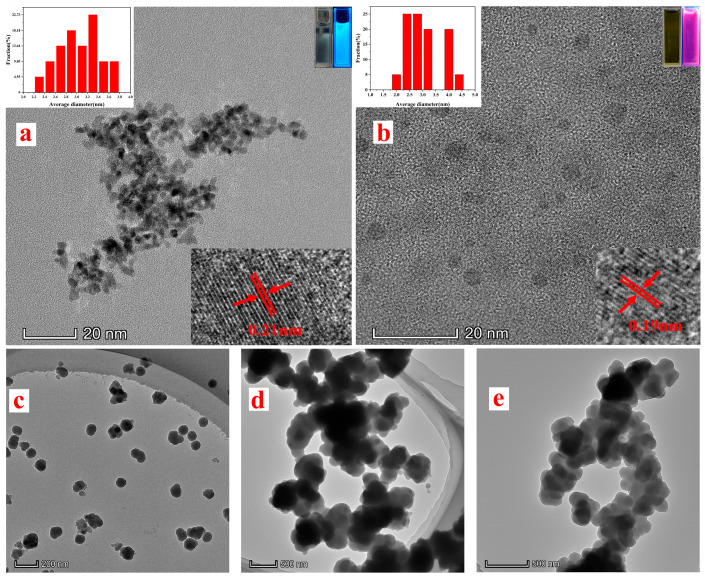
TEM images for (**a**) BCDs, (**b**) RCDs and (**c**) RCDs@SiO_2_, (**d**) MIPs@BCDs/RCDs@SiO_2_ and (**e**) NIPs@BCDs/RCDs@SiO_2_, with insets for particle size diagrams (upper left of (**a**,**b**)), ultraviolet and visible diagrams (upper right of (**a**,**b**)), and lattice fringe diagrams (lower right of (**a**,**b**)).

TEM observed the particle sizes and morphologies of BCDs and RCDs. Figure 1a,b shows that both BCD and RCD particles are nearly spherical and well-dispersed, with an average diameter of approximately 3 nm. The lattice fringes of BCDs and RCDs are visible in the HRTEM image (inset of Figure 1a,b). In addition, BCDs emitted bright blue visible light, while RCDs emitted bright red visible light when excited by ultraviolet light at a wavelength of 365 nm. Figure 1c shows that the particle size distribution of RCDs@SiO_2_ was uniform and that the particle diameters ranged from 86 to 152 nm. After the MIPs and BCDs were grafted, the synthesized MIPs@BCDs/RCDs@SiO_2_ (Figure 1d) was 2–3 times the size of RCDs@SiO_2_. This meant that MIPs@BCDs/RCDs@SiO_2_ showed high cross-linking ability between Si-O-Si chains, providing a stable, rigid structure. The morphology of NIPs@BCDs/RCDs@SiO_2_ (Figure 1e) was similar to that of MIPs@BCDs/RCDs@SiO_2_, but the outer surface of the latter appeared rougher, confirming the formation of imprinted cavities in MIPs due to the presence of the dummy templates during polymerization.

FT-IR was used to determine the specific chemical groups found on BCDs, RCDs, MIPs@BCDs/RCDs@SiO_2_, and NIPs@BCDs/RCDs@SiO_2_. Figure S1 shows the spectrum for MIPs@BCDs/RCDs@SiO_2_; the band at 1388–800 cm^−1^ was attributable to Si-O-Si and Si-O stretching vibration. The peak at 459 cm^−1^ represented Si-O-C bending vibration. The characteristic peak signals at 3000–3500 cm^−1^ were ascribed to the stretching vibration of hydroxyl groups and amino groups. Compared with BCDs and RCDs, MIPs@BCDs/RCDs@SiO_2_ has a spectrum with larger characteristic peak areas of Si-O-Si and Si-O bonds, as well as new absorption peaks representing Si-O-C bonds. All the above information indicates the successful introduction of APTES or TEOS in polymerization. The stretching vibrations of C=O and C=C at 1692 cm^−1^ were in the spectrum, clearly confirming the existence of CDs in MIPs@BCDs/RCDs@SiO_2_.

To further ascertain the synthesis of MIPs@BCDs/RCDs@SiO_2_ and its elemental composition, XPS spectra were measured, with results shown in Figure S2. The absorption peaks in the C1s map at 287.8 eV, 285.6 eV, and 284.7 eV were attributable to C-N/C=O, C-O, and C=C/C-C, respectively. The 402.7 eV and 399.3 eV absorption peaks in the N1s map belonged to C-N and N-O, respectively. The 535 eV and 532.1 eV absorption peaks in the O1s map were attributable to C-OH/C-O-C and C=O, respectively. The 104.7 eV and 101.8 eV absorption peaks in the Si2p map belonged to Si-C and Si-O, respectively. Thus, the XPS analysis results for MIPs@BCDs/RCDs@SiO_2_ were generally consistent with the FT-IR results.

### 3.2. Optical Properties

As observed in the UV–Vis absorption spectra in Figure S3, the two distinct characteristic absorption peaks exhibited by BCDs under 360 nm excitation were at 222 nm and 356 nm, attributable to π–π* transitions of C=C bonds and n–π* transitions of C=O bonds, respectively. Among these, C=C bonds might derive from pyrolysis of anhydrous citric acid, leading to intramolecular dehydration. RCDs showed four distinct characteristic absorption peaks due to various complex surface functional states: 259 nm, 308 nm, 408 nm, and 666 nm. The peak at 259 nm was attributable to conjugated π–π* transitions of C=C bonds. The absorption peaks at 308 nm and 408 nm were ascribed to n–π* transitions of C=O bonds and C=N bonds. The absorption peak at 666 nm might be due to some trace metals in Osmanthus leaves, leading to charge transfer between metal–ligand bonds [37].

The best excitation wavelengths for RCDs and BCDs were obtained by fluorescence spectrometer characterization. As shown in Figure S4a, BCDs and RCDs exhibited maximum fluorescence intensities centered at 440 and 675 nm, respectively, when the excitation wavelength was 360 nm. BCDs’ directly calculated relative quantum yield was 51% (Figure S4b) using quinine sulfate as a standard sample (54% in 0.1 mol/L). Due to the particularity of the fluorescence emission spectrum of RCDs, it was difficult to find a matching reference reagent to calculate the relative quantum yield. Therefore, the absolute quantum yield of RCDs was measured by a steady-state, transient fluorescence spectrometer. As shown in Figure S4c, the measurement result was 12%, indicating that the prepared RCDs showed suitable fluorescence emission ability to be used as the reference signal of a ratiometric fluorescence sensor.

### 3.3. Optimization of Fluorescent Detection Conditions

**Figure 2:**
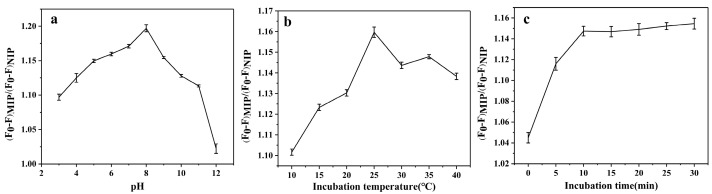
Effects of (**a**) pH value, (**b**) incubation temperature, and (**c**) incubation time on fluorescence quenching efficiency (*n* = 3). Experimental conditions: V_MIPs@BCDs/RCDs@SiO__2_ = 100 μL, λ_ex_ = 360 nm, C_P-CTX-3C_ = 0.1 ng/mL.

The most important factors affecting the detection performance of the fluorescence sensor were pH value, incubation temperature, and incubation time. As shown in Figure 2a, the PL capacity approached the maximum at a pH of 8, indicating that the optimum fluorescence quenching efficiency was obtained in an alkaline medium. This was probably due to the protonation and deprotonation of functional groups on the carbon dot surfaces [38].

Figure 2b presents the effect of incubation temperature on fluorescence quenching. The incubation temperature was set in the range of 10–40 °C with gradient increments of 5 °C. The optimum fluorescence quenching efficiency was obtained when the reaction temperature reached 25 °C. This can be explained as follows: the molecules did not have enough energy to form bonds when the temperature was low. However, the molecular energy was high when the temperature was high, and the intensification of thermal motion was not conducive to bonding. 

Incubation time was another key factor. As shown in Figure 2c, the fluorescence quenching efficiency increased rapidly over the first 10 min of incubation time, finally reaching equilibrium in the range of 10–30 min. This finding shows that the adsorption of the recognition sites reached saturation within 10 min. In all, these results show that the best fluorescence quenching performance was obtained at a solution pH of 8, an incubation temperature of 25 °C, and an incubation time of 10 min.

### 3.4. Fluorescence Detection

**Figure 3:**
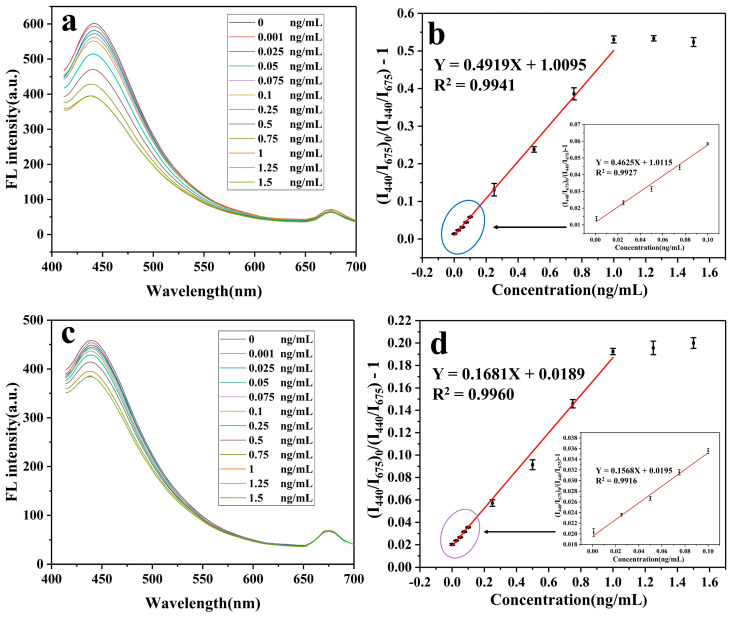
(**a**,**c**) Fluorescence quenching diagrams and (**b**,**d**) Stern–Volmer curves for (**a**,**b**) MIPs@BCDs/RCDs@SiO_2_ and (**c**,**d**) NIPs@BCDs/RCDs@SiO_2_.

The adsorption performance and fluorescence quenching performance of MIPs@BCDs/RCDs@SiO_2_ were further examined under optimal conditions. As displayed in Figure 3a, when MIPs@BCDs/RCDs@SiO_2_ was excited at 360 nm, the fluorescence intensity at 440 nm regularly decreased with the increasing addition of P-CTX-3C in the range of 0–1.0 ng/mL. The fluorescence intensity at 675 nm remained unchanged. The Stern–Volmer equation *F*_0_/*F* = 1 + [*C*]·*K_SV_* was used to establish the correlation curve between the concentration of P-CTX-3C and the corresponding quenching fluorescence intensity over a certain concentration range. Here, *F_0_* and *F* represent the fluorescence intensity in the absence and presence of P-CTX-3C, namely (I_440_/I_675_)_0_ and I_440_/I_675_. *K_SV_* is the quenching constant of P-CTX-3C, and [*C*] represents the concentration of P-CTX-3C. A linear correlation was found between *F*_0_/*F* and the concentration of P-CTX-3C over the concentration range from 0.001 to 1.0 ng/mL. The corresponding regression equation was y = 0.4919 x + 0.0095, with a coefficient of determination (R^2^) of 0.9941 (Figure 3b).

For comparison, the fluorescence response of NIPs@BCDs/RCDs@SiO_2_ to P-CTX-3C was also examined. Figure 3c shows that the fluorescence intensity at 440 nm changed slightly after the addition of the same CTX3C concentration range tested in the trial described above, due to the absence of tailor-made binding sites in NIPs@BCDs/RCDs@SiO_2_. Therefore, the possible interaction between CTX3C and its binding site was reduced. Consequently, the degree of fluorescence quenching was lower. Furthermore, the imprinting factor (IF), defined as the ratio of *K*_SV,MIPs_ to *K*_SV,NIPs_, was used to evaluate the molecular recognition ability and fluorescence sensing performance of MIPs@BCDs/RCDs@SiO_2_. Generally, the higher the value of IF, the stronger the selectivity of imprinted polymer, and the better the recognition and sensing effect. The IF value was determined to be 2.93, indicating that a stronger sensing system from molecular recognition to fluorescence quenching was built between P-CTX-3C and MIPs@BCDs/RCDs@SiO_2_ than between P-CTX-3C and NIPs@BCDs/RCDs@SiO_2_. 

In addition, 3σ/K was used to calculate the detection limit of MIPs@BCDs/RCDs@SiO_2_, where K is the slope of the best-fitting linear relationship, and σ is the standard deviation of the fluorescence intensity value of the blank sample averaged over its 11 replicate tests. The lower limit of detection was calculated to be 3.3 × 10^−4^ ng/mL, suggesting the possibility of the developed sensor for trace detection of P-CTX-3C.

### 3.5. Selectivity and Anti-Interference Ability of the Sensor

**Figure 4:**
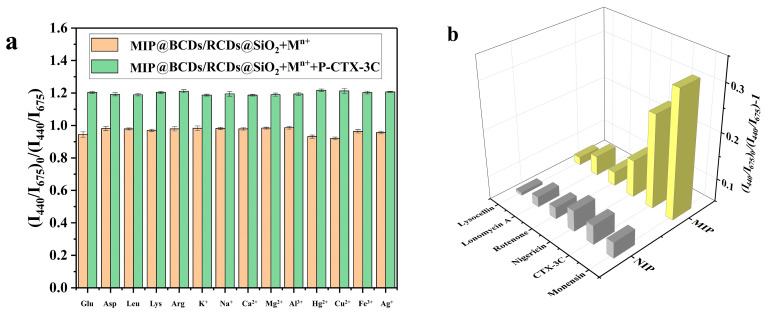
(**a**) Interference effects of various amino acids and metal ions; (**b**) selectivity diagram for MIPs@BCDs/RCDs@SiO_2_ and NIPs@BCDs/RCDs@SiO_2_.

Selectivity, one of the prominent advantages of molecular imprinting, is based on the existence of recognition sites in the polymer structure that are complementary to the template in terms of size, shape, and positioning of chemical groups. In order to assess the selectivity of the ratiometric fluorescence sensor, six structural analogs (monensin, P-CTX-3C, nigericin, rotenone, ionomycin A, and lysozyme; molecular formulas displayed in Figure S5) were absorbed by MIPs@BCDs/RCDs@SiO_2_ and NIPs@BCDs/RCDs@SiO_2_. The fluorescence quenching intensity *F*_0_/*F* was examined and analyzed. The larger the *F*_0_/*F* value, the stronger the fluorescence quenching intensity and the better the adsorption effect. As seen in Figure 4a, monensin showed the best quenching effect among all the structural analogs of MIPs@BCDs/RCDs@SiO_2_, followed closely by P-CTX-3C, with the rest of the analogs performing poorly. The structure of monensin is similar to the fragmentary structure of P-CTX-3C. Accordingly, many recognition sites were generated during the imprinting process to specifically rebind with monensin and P-CTX-3C, quenching the fluorescence of BCDs. In contrast, the other analogs with different shapes and molecular weights were not complementary to the recognition sites and could not effectively influence the fluorescence intensity of BCDs. For NIPs@BCDs/RCDs@SiO_2_, the quenching of fluorescence intensity was almost negligible at the same concentrations of P-CTX-3C and its analogs, revealing that NIPs@BCDs/RCDs@SiO_2_ did not show significant selectivity for these analogs.

Additionally, a variety of amino acids and metal ions with a concentration of 0.2 μmol/L, including glucose (Glu), aspartate (Asp), leucine (Leu), lysine (Lys), arginine (Arg), K^+^, Na^+^, Ca^2+^, Mg^2+^, Al^3+^, Hg^2+^, Cu^2+^, Fe^3+^, and Ag^+^, were selected as the interfering factors in the detection process. As shown in Figure 4b, none of the above amino acids and metal ions significantly impacted the quenching of fluorescence intensity. This outcome may be due to the negligible interference effect caused by amino acids and metal ions compared with the quenching intensity of the sensor caused by P-CTX-3C. The results show that the fluorescence sensor had strong internal self-tuning ability and excellent anti-interference ability (i.e., the ability of fluorescence sensor to confront the interfering substances including amino acids and metal ions) in a complex matrix.

### 3.6. Reproducibility of the Sensor

To investigate the reproducibility of MIPs@BCDs/RCDs@SiO_2_, four cycles of absorption and desorption of P-CTX-3C were carried out. As displayed in Figure S6, after four cycles of absorption and desorption, the average fluorescence quenching efficiency of the MIPs@BCDs/RCDs@SiO_2_ was 75.16%. On one hand, since some structures would inevitably be damaged, the effective imprinting sites reduced. On the other hand, a part of BCDs might be lost, resulting in a certain degree of weakening of the overall fluorescence intensity. Therefore, within the four cycles, though the process would destroy the molecular recognition system and fluorescence sensing system to a certain extent, the average fluorescence quenching efficiency reached approximately 75%, indicating that the fluorescence sensor possessed excellent reproducibility.

### 3.7. Analysis of Fishes Spiked with CTX 

To demonstrate the applicability of the developed method to the analysis of natural samples, three species of fish with potential ciguatera poisoning, namely eel, bass, and grouper, were selected. The samples were pretreated as described in Section 2.7 and the filtrate was tested with the described method and with LC/MS. 

Table 1 summarizes the results for the spiked and non-spiked samples. The average recovery of P-CTX-3C varied from 80.00% to 91.33%, with a relative standard deviation (RSD) of less than 9.91%, indicating the feasibility of the as-prepared sensor for trace P-CTX-3C determination in real-world complex samples. Moreover, all the results obtained by this method are consistent with the LC-MS results.

According to the results, P-CTX-3C was not detected in the three kinds of medium and large coral reef fish. This might be due to the fact that the *Gambierdiscus* algae ingested by the samples contained merely ciguatoxin precursors with a low degree of oxidation in vivo, causing the precursors of ciguatoxin to fail to convert into P-CTX-3C with strong toxicity given the weak polarity and low toxicity. This resulted in the undetected cases in real samples.

Table 2 compares our developed method and other previously reported methods for P-CTX-3C detection. When comparing the fluorescent sensor with LC-MS, both methods use the principle of identifying the structure of toxins to detect. The fluorescent sensor can quickly identify the structure of P-CTX-3C by means of virtual template imprinting and achieve the quantitative analysis of toxins through the quenching of fluorescence intensity, which has the advantages of high sensitivity and high selectivity. For the reported LC-MS methods used by the China National Standard and Sibat et al., the LC-MS methods can specifically and quickly identify the structure and quantitative analysis of different toxins. It can also provide information on toxin profiles. Nevertheless, compared to the low LOD of fluorescent sensor of 3.3 × 10^−4^ ng/mL, LC-MS methods are less sensitive, with LOD of 0.01 ng/mL [7,39].

Fluorescent sandwich ELISA and the fluorescence sensor have similar recognition principles. The difference is that fluorescent sandwich ELISA recognizes the toxin structure of P-CTX-3C through antibodies, while the fluorescence sensor recognizes toxin structure through virtual molecularly imprinted polymers. For fluorescent sandwich ELISA conducted by Tsumuraya et al., adding immunoreaction enhancer solution (Can Get Signal^®^) to the fluorescent sandwich ELISA system, the LOD was as low as 9.0 × 10^−5^ ng/mL and the background fluorescence was significantly lowered. Nevertheless, compared to the fluorescence sensor, it has a relatively long detection time of 2.5 h because of the complex operation steps [40].

Some creative and practical methods have been recently established, including fluorescent receptor binding assay, electrochemical biosensors, and an immunosensor controlled by a smartphone. The readily available and most widely functional assay is known as the cell-based assay (CBA), and the neuroblastoma (N2a) cell line appears as the most promising one. When comparing the fluorescence sensor and the CBA, they differ in detection principles. The CBA quantifies the amount of P-CTX-3C by measuring the cytotoxic effect it has on N2a cells, while the fluorescence sensor detects the presence of the toxin according to its structure. When applied for the detection of P-CTX-3C, the LOD value is 0.031 ng/mL and the detection time is 3 h [41]. Another CBA method conducted by Loeffler et al. achieved the sensitivity and specificity of the assay to compounds activating the NaV through the addition of the pharmaceuticals ouabain (O) and veratridine (V). The LOD of P-CTX-3C reaches 1.35 × 10^−3^ ng/mL [42]. Compared to the CBA methods, the fluorescence sensor has the advantages of lower detection limit and shorter detection time. 

The electrochemical biosensor and fluorescence sensor are partially similar in recognition. For the electrochemical biosensor, three different mAbs (3G8, 10C9, and 8H4) that specifically bind to one of the wings of P-CTX-3C are used to develop a sandwich immunosensor. Magnetic beads (MBs) are exploited as a support to provide an enlarged surface area for the immobilization of mAbs, to shorten the analysis time, and to minimize matrix effects. Imprinted holes of the fluorescence sensor are used to specifically bind to the partial structure of P-CTX-3C to induce the quenching of fluorescence. For the electrochemical biosensor, the LOD value of 3.59 × 10^−3^ ng/mL is obtained for P-CTX-3C and the detection time is 2.1 h [43].

As for the immunosensor controlled by a smartphone, the detection principle is basically the same as the electrochemical biosensor mentioned above. The improvement is that captured mAbs were immobilized on multiwalled carbon nanotube (MWCNT)-modified carbon electrodes instead of magnetic beads. The sandwich assay was then conducted, and amperometric signals were measured with a smartphone-controlled potentiostat. The LOD value is 1.0 × 10^−3^ ng/mL for CTXs using highly specific and sensitive mAbs and the detection time is 2 h [44].

Radioligand receptor binding assays (r-RBA) rely on the use of a brain membrane preparation rich in voltage-gated sodium channels, the pharmacological receptors of CTXs. The CTXs in a sample compete with a radiolabeled toxin to bind to these shared receptors, available in a reliable high-throughput microplate format that quantitatively reports the composite toxic potency of seafood samples containing a mix of different CTX analogues [6]. The detection principles differ between r-RBA and the fluorescence sensor. Compared to the fluorescence sensor, this optimized r-RBA can detect P-CTX-3C concentrations at 0.75 ng/mL with the detection time of 3 h.

In our study, we obtained a minimum lower LOD of 3.3 × 10^−4^ ng/mL, which means that even trace P-CTX-3C could be sensitively recognized. Furthermore, the response speed of this work is fast—the time from the end of sample pretreatment to results is only 15 min. Last but not least, the fluorescence sensor preparation was cost-effective. For LOD, since the calculation formula of LOD is 3σ/K, it could be analyzed from the following aspects. Firstly, because the signal acquisition mechanism of the fluorescence sensor used the ratiometric analysis of RCDs as reference signals and BCDs as response signals, the reference signals and response signals changed synchronously with the change in the influencing factors of the external environment. Therefore, the sensor had strong self-correction ability and stability, making the standard deviation of detection a small value of σ. Secondly, because the response signals BCDs outside the sensor had excellent quantum efficiency, it was sufficient to produce a sharp fluorescence quenching effect on the energy change (i.e., the system energy change caused by MIPs adsorption of P-CTX-3C), resulting in a higher value of linear relationship curve slope of K. Therefore, we obtained a lower LOD of 3.3 × 10^−4^ ng/mL in the study. For detection time, it could be analyzed from the structure. Because the MIPs were prepared by the surface imprinting method, the imprinted sites on the surface could absorb P-CTX-3C faster and affect the response signal. The overall sensor adopted the preparation method of a core–shell structure, so the external response signal BCDs had high quenching efficiency, which could respond efficiently and quickly produce a strong quenching effect. Therefore, this study could obtain a faster detection time of 15 min. For preparation cost, the raw materials for preparing CDs, MIPs, and the fluorescence sensor, and the instruments and equipment used for detection and analysis, were generally economical. Therefore, the fluorescence sensor preparation was cost-effective compared to other methods.

Notably, we did not conduct the determination of the most representative ciguatoxin P-CTX-1B due to the lack of standard analyte. When compared with other individual methods which can simultaneously determinate P-CTX-3C and P-CTX-1B, this is inevitably a regrettable deficiency. Nevertheless, we believe that the sensor can realize the simultaneous determination of two or more toxins with appropriate adjustments. Additionally, there was no analysis of naturally contaminated samples because they were difficult to obtain. In conclusion, the integrity of the study will be improved with appropriate adjustments.

Taken altogether, though there are advantages and disadvantages in P-CTX-3C analysis, the proposed ratiometric fluorescence sensors promise P-CTX-3C determination in complex samples in terms of sensitivity, quick response, low cost, and practicability.

## 4. Conclusions

We have presented the quick construction of a dual-emitting ratiometric fluorescence sensor using CDs as the MIP response signal and reference signal materials for P-CTX-3C determination. By integrating the sensitivity of ratiometric fluorescence methods and the selectivity of MIPs, the MIPs@BCDs/RCDs@SiO_2_ sensor can specifically recognize and rapidly analyze trace P-CTX-3C in coral reef fish meat. Moreover, due to the innovativeness and convenience of the fluorescence sensor, it requires no sophisticated or expensive instrumentation or complicated training to perform the analysis. The MIPs sensor possessed excellent reusability. After four cycles of absorption and desorption, the average fluorescence quenching efficiency remained about 75%. This capability will facilitate important decision-making steps and reduce the risk of human exposure to toxins. Moreover, a novel fragmentary dummy template method for preparing MIPs was proposed herein, paving the way for new means of detecting larger molecules with complex structures.

## Figures and Tables

**Table 1:** MIPs@BCDs/RCDs@SiO_2_ and LC/MS determination results for P-CTX-3C in fish.

Samples	Added (ng/mL)	Found in this Work (ng/mL)	Found in LC/MS (ng/mL)	Average Recovery (%)	RSD (%, *n* = 3)
Eel	0.000	ND ^a^	ND ^a^	-	-
	0.025	0.020 ± 0.004	0.021 ± 0.003	80.00	9.91
	0.250	0.211 ± 0.010	0.203 ± 0.020	84.40	8.52
	0.750	0.685 ± 0.020	0.701 ± 0.030	91.33	7.71
Bass	0.000	ND ^a^	ND ^a^	-	-
	0.025	0.020 ± 0.002	0.021 ± 0.004	80.00	9.62
	0.250	0.213 ± 0.030	0.221 ± 0.010	85.20	7.50
	0.750	0.663 ± 0.040	0.706 ± 0.030	88.40	6.81
Grouper	0.000	ND ^a^	ND ^a^	-	-
	0.025	0.021 ± 0.003	0.021 ± 0.005	84.00	9.20
	0.250	0.207 ± 0.010	0.222 ± 0.030	82.80	5.62
	0.750	0.679 ± 0.020	0.713 ± 0.020	90.53	7.93

^a^ Not detectable.

**Table 2:** Comparison of representative analytical methods for P-CTX-3C detection.

Samples	Method	LOD (ng/mL)	Detection Time	References
Aquatic products	LC-MS	0.10	30 min	[39]
*Tripneustes gratilla,* etc.	LC-MS/MS	0.01	30 min	[7]
*Variola louti,* etc.	Sandwich ELISA	9.0 × 10^−5^	2.5 h	[40]
Aquatic products	Neuroblastoma cell-based assay	0.031	3 h	[41]
*Lutjanus malabaricus*	Neuroblastoma cell-based assay	1.35 × 10^−3^	>1 day	[42]
*Variola louti,* etc.	Electrochemical biosensor	3.59 × 10^−3^	2.1 h	[43]
*L. bohar,* etc.	Immunosensor controlled by a smartphone	1.0 × 10^−3^	2 h	[44]
Barracuda, etc.	Radioligand receptor binding assay	0.75	3 h	[6]
Coral reef fish meat	Molecularly imprinted fluorometry	3.3 × 10^−4^	15 min	This work

## Data Availability

The data are contained within the article.

## Supplementary Materials

The following supporting information can be downloaded at: https://www.mdpi.com/article/10.3390/foods11203239/s1, Figure S1: FT-IR diagram of RCDs, BCDs (a) and MIPs@BCDs/RCDs@SiO_2_, NIPs@BCDs/RCDs@SiO_2_ (b); Figure S2: XPS spectra of MIPs@BCDs/RCDs@SiO_2_ and C1s (a), N1s (b), O1s (c) and Si2p (d); Figure S3: UV–Vis diagram of RCDs and BCDs; Figure S4: Fluorescence spectra of RCDs and BCDs (a), linear curve comparison of BCDs and quinine sulfate (b), quantum yield of RCDs (c); Figure S5: Molecular formulas of P-CTX-3C (a), monensin (b), nigericin (c), rotenone (d), ionomycin A (e), and lysozyme (f); Figure S6. Four rounds of absorption and desorption of P-CTX-3C.
