# Supplementary material for: Facile Synthesis of Molecularly Imprinted Ratiometric Fluorescence Sensor for Ciguatoxin P-CTX-3C Detection in Fish
Source: Foods. 2022 Oct 17;11(20):3239. doi: 10.3390/foods11203239 (PMC9601512; doi:10.3390/foods11203239)
Supplement: Supplementary file 1 [file foods-11-03239-s001.zip › foods-1929986-supplementary.pdf]

Supporting information

Facile Synthesis of Molecularly Imprinted Ratiometric Fluorescence Sensor for Ciguatoxin P-CTX-3C Detection in Fish

Zhenke Qi, Cheng Xiang, Xingguo Tian and Xiaoyan Xu*

Guangdong Provincial Key Laboratory of Food Quality and Safety, College of Food Science, South China Agricultural University, Guangzhou 510642, China

* Correspondence: yanzixu_2001@scau.edu.cn; Tel.: +86-020-85283448

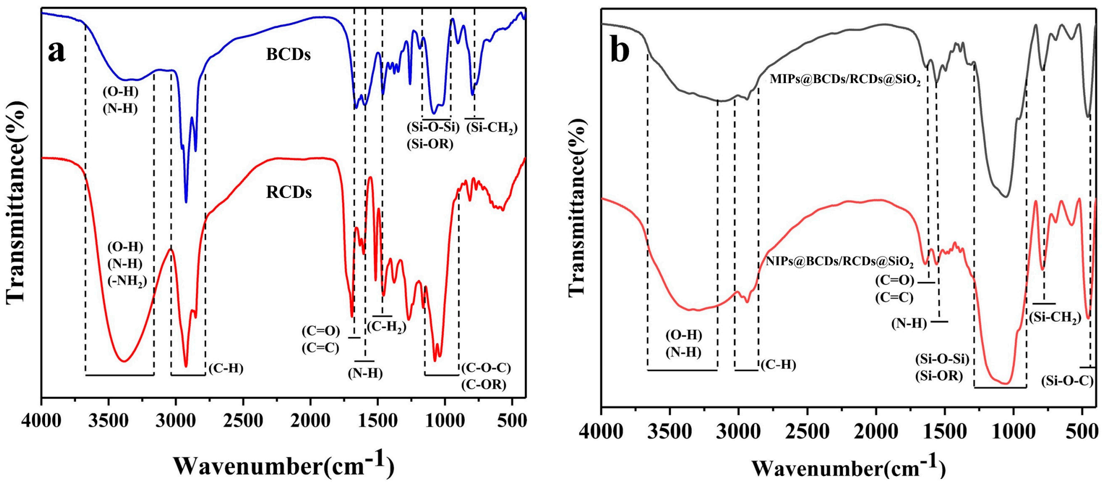

Figure S1. FT-IR diagram of RCDs, BCDs(a) and MIPs@BCDs/RCDs@SiO_2, NIPs@BCDs/RCDs@SiO_2(b)

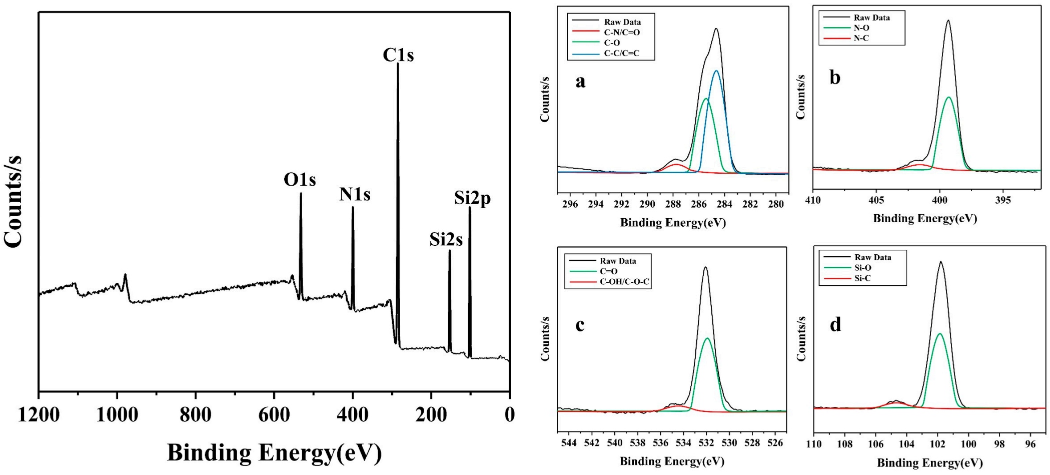

Figure S2. XPS spectra of MIP@BCDs/RCDs@SiO_2 and C1s(a), N1s(b), O1s(c) and Si2p(d)

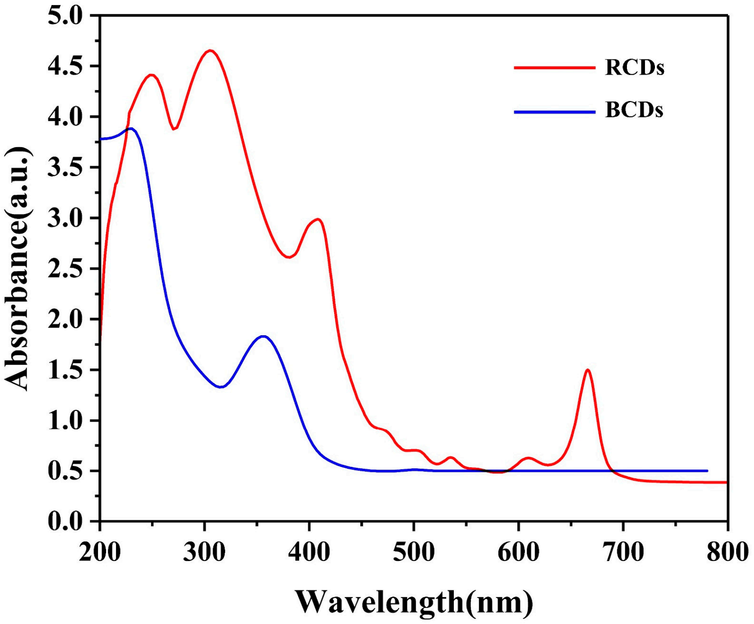

Figure S3. Uv-vis diagram of RCDs and BCDs

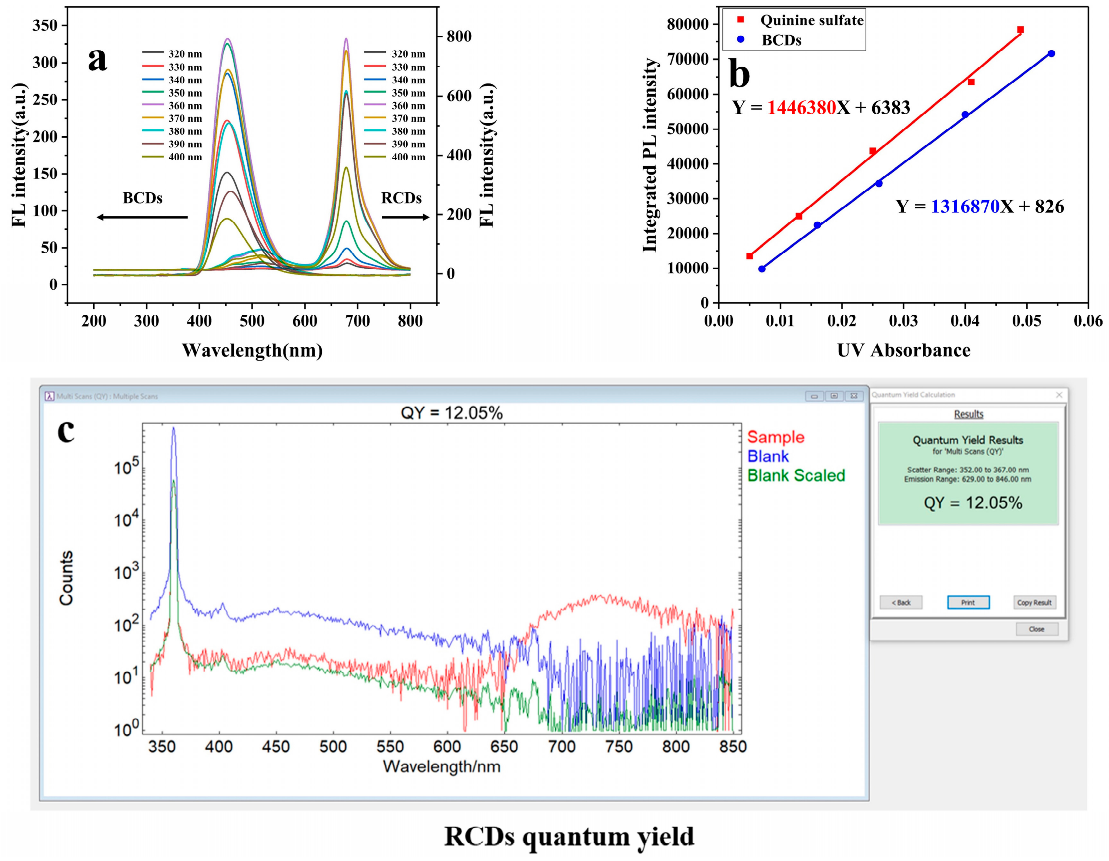

Figure S4. Fluorescence spectra of RCDs and BCDs(a), linear curve comparison of BCDs and quinine sulfate(b), quantum yield of RCDs(c)

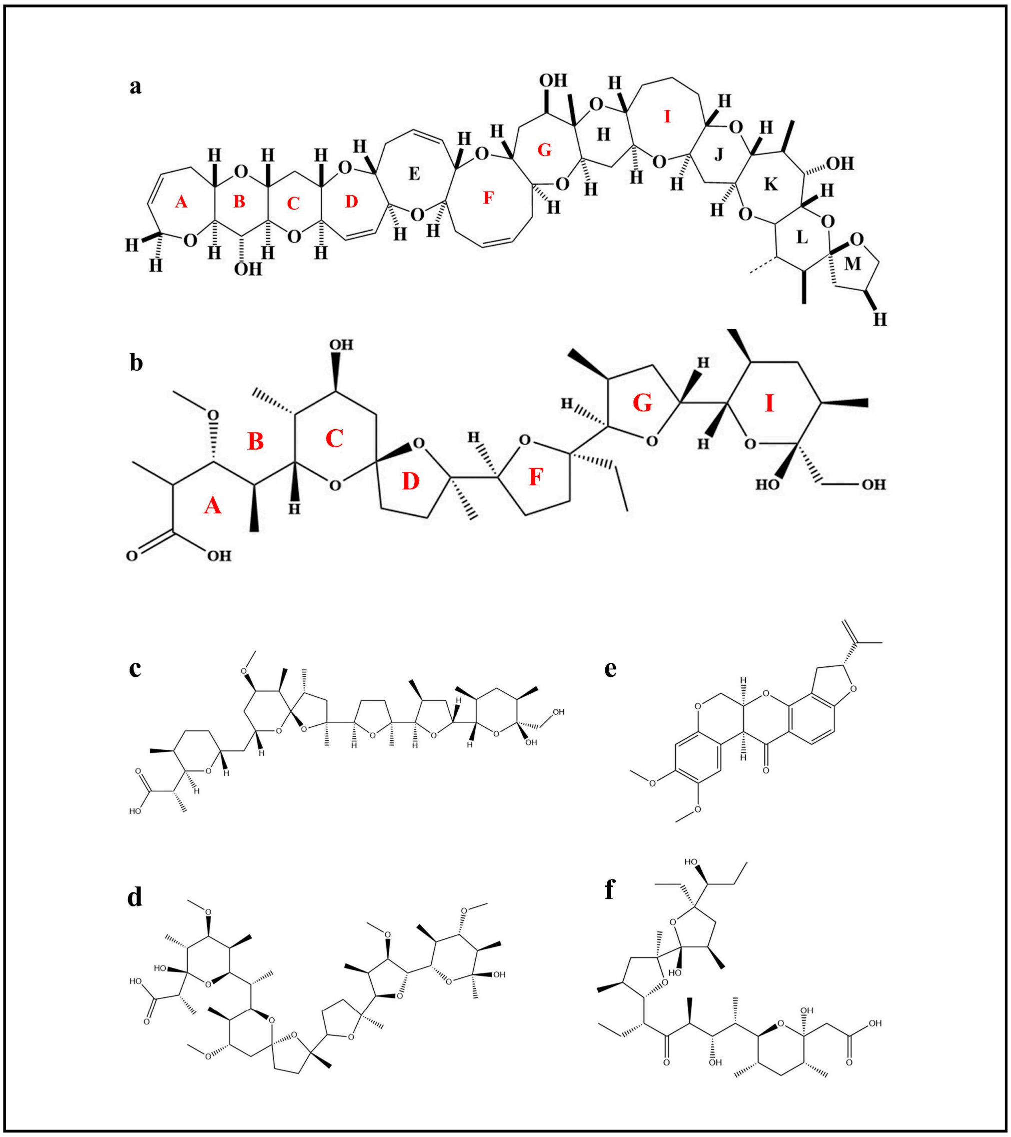

Figure S5. Molecular formulas of P-CTX-3C(a), monensin(b), nigericin(c), rotenone(d), ionomycin A(e) and lysozyme(f)

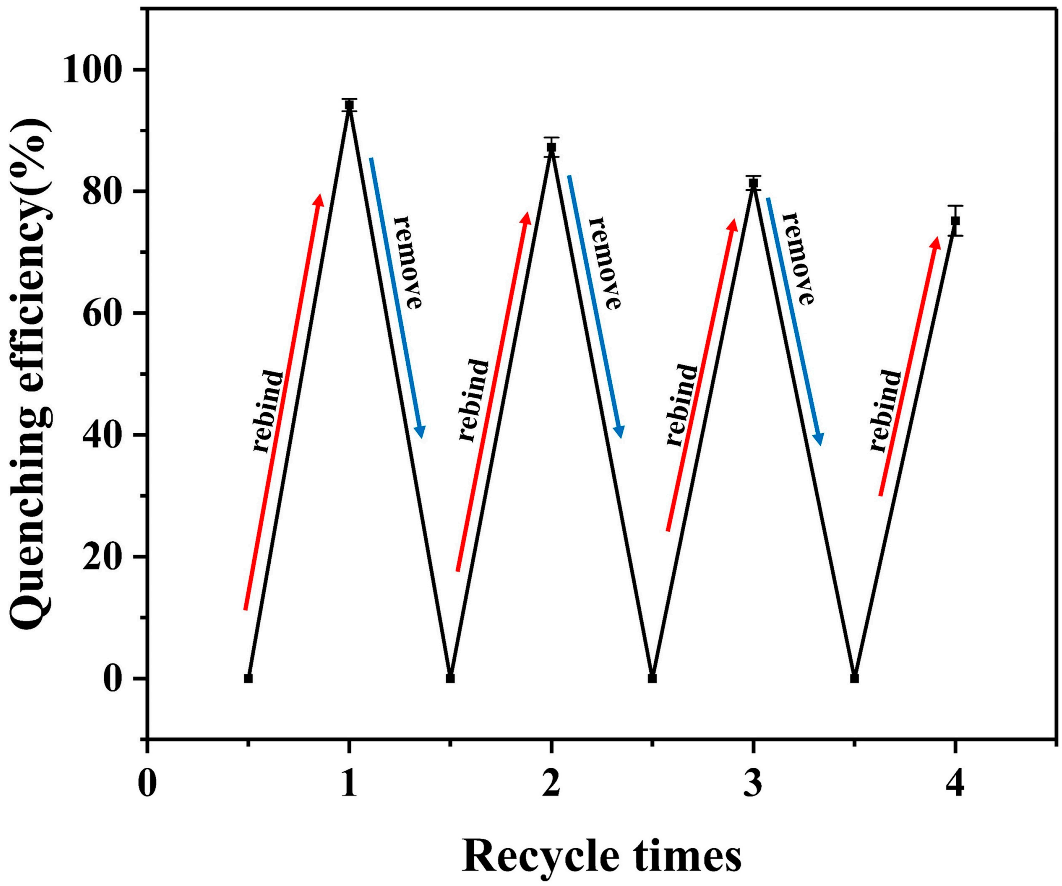

Figure S6. Four cycles of absorption and desorption of P-CTX-3C
